# Constant Versus Ascending Radial Pressure Wave Therapy for Pain Management in Carpal Tunnel Syndrome: A Pilot Exploratory Study

**DOI:** 10.3390/jcm15218209

**Published:** 2026-10-22

**Authors:** Armando Tonatiuh Ávila-García, Ana Lilia Villagrana-Rodríguez, Elba Janette Ledezma-Miramontes, Emmanuel Mercado-Nuñez, Martha Alejandra García-Casillas, Erick José Ontiveros-Castellanos, Karen Chacón-Morales, Daniel Miller-Serrano, Diana Hazel Araiza-Quintana, Luis Fernando Herrera-Fuentes, Judith Carolina De Arcos-Jiménez, Jaime Briseno-Ramirez

**Affiliations:** 1 Servicio de Medicina Física y Rehabilitación, Hospital Civil de Guadalajara Fray Antonio Alcalde, Guadalajara 44200, Mexico; atavila@hcg.gob.mx (A.T.Á.-G.); alvillagrana@hcg.gob.mx (A.L.V.-R.); ejledezma@hcg.gob.mx (E.J.L.-M.); emercadonunez@hcg.gob.mx (E.M.-N.); mgarciac@hcg.gob.mx (M.A.G.-C.); eontiveros@hcg.gob.mx (E.J.O.-C.); karen.chm2662@gmail.com (K.C.-M.); green.miller26@gmail.com (D.M.-S.); hazel.araiza@alumnos.udg.mx (D.H.A.-Q.); 2 Centro Universitario de Tlajomulco, Universidad de Guadalajara, Tlajomulco de Zúñiga 45641, Mexico; judith.dearcos@academicos.udg.mx; 3 Hospital Civil de Oriente, Tonalá 45425, Mexico; lfherrera@hcg.gob.mx; 4 Centro Universitario de Tonalá, Universidad de Guadalajara, Av. Nuevo Periférico No. 555, Ejido San José Tateposco, Tonalá 45425, Mexico

**Keywords:** carpal tunnel syndrome, radial pressure wave therapy, pilot study, feasibility, pain management, rehabilitation, exploratory study

## Abstract

**Background/Objectives:** This exploratory report compares two radial pressure wave therapy (RPWT) regimens for carpal tunnel syndrome and describes clinical trajectories and observations relevant to future trial design. The statistical plan and the selection of the 2-month comparison followed inspection of the study data. **Methods:** Fifty-one participants were randomized (25 constant, 26 ascending; NCT07070661). Seven left the study (six ascending, one constant); forty-three contributed clinical scores. Both arms received three weekly sessions and the same home exercise program; pressure, frequency, and theoretical emission duration differed jointly. The main exploratory comparison was between-regimen pain change at 2 months. **Results:** The pain difference (ascending minus constant) was −0.54 points on the numeric pain rating scale (NPRS, 0–10; 95% CI −2.14 to 1.06), and observed mean NPRS decreased from baseline to 2 months in both arms: from 6.50 to 4.04 in the constant arm and from 6.37 to 3.37 in the ascending arm. Descriptive observations included asymmetric attrition (six of seven departures in the ascending arm) and observed baseline imbalance (grip strength SMD 0.624; symptom duration 28.7 versus 39.2 months). Three participants had early indications for carpal tunnel release. **Conclusions:** Although mean pain improved in both groups, these non-conclusive pilot data establish neither superiority nor equivalence. The observed improvement cannot be attributed to the RPWT device because both groups received exercises and no sham or exercise-only control was included. These findings inform a definitive trial with stratified randomization and an appropriate comparator.

## 1. Introduction

Carpal tunnel syndrome (CTS) is the most common entrapment neuropathy of the upper limb [1]. Population estimates distinguish symptoms in the median nerve distribution from clinically and electrophysiologically confirmed disease [2]. Nocturnal paresthesiae, pain, and sensory loss can impair sleep, dexterity, and daily functioning. Difficulty manipulating small objects and performing repetitive or precision hand tasks can restrict work capacity and quality of life; advanced disease may include thenar weakness and atrophy [1].

Conservative options investigated for mild-to-moderate CTS include night splinting, corticosteroid injection, manual therapy, and transcutaneous electrical nerve stimulation (TENS). Evidence supporting these interventions differs, and investigation of TENS does not establish superiority over established conservative care [3]. Radial pressure wave therapy (RPWT) uses pneumatically generated, radially propagating pressure waves. It is physically distinct from focused extracorporeal shockwave therapy, although reviews sometimes group the modalities together [4].

Reviews of shockwave-based treatments for CTS have reached different conclusions, reflecting heterogeneous interventions, comparators, and methods [5,6]. An umbrella review judged the evidence for shockwave therapy inconclusive and identified substantial methodological weaknesses in the underlying reviews [7]. Previous trials investigated session number, pressure settings, and impulse count [8,9,10]. These questions differ from comparing a constant regimen with an ascending pressure–frequency regimen when total impulses and the nominal pressure–impulse product are equal. Such nominal matching does not establish acoustic energy equivalence.

CTS severity can be described using conventional electrophysiological grading or investigated using predictive approaches. Recent machine-learning studies have examined postoperative electrophysiological and questionnaire outcomes and explainable severity classification using clinical data [11,12]. These methods address prediction or classification; they do not by themselves validate causal treatment comparisons or replace the clinical interpretation of nerve conduction findings.

Pilot and feasibility studies can inform future trial design through recruitment, retention, and variability estimates; prospective feasibility objectives and progression criteria distinguish such studies from efficacy comparisons [13,14]. The pilot exploratory designation emphasizes the study’s contribution to planning a future definitive trial through descriptive information on retention, outcome completeness, tolerability, and outcome variability.

The objectives of this report are to describe retention, available tolerability observations, and baseline prognostic imbalance, and to present exploratory between-regimen differences in pain intensity following constant or ascending pressure–frequency RPWT, each accompanied by a common home exercise program. BCTQ symptom severity and functional status are secondary exploratory outcomes. The comparison concerns complete regimens because pressure, frequency, and theoretical emission duration differed jointly.

## 2. Materials and Methods

### 2.1. Study Design and Setting

This two-arm study was conducted at the Department of Physical Medicine and Rehabilitation, Antiguo Hospital Civil de Guadalajara “Fray Antonio Alcalde”, Guadalajara, Mexico, with participants randomized to one of two complete RPWT regimens. The study is reported as a pilot exploratory comparison to inform future trial design through descriptive process measures and estimates of outcome variability. Feasibility objectives and progression criteria were not prespecified; the original sample-size calculation is retained below. The ClinicalTrials.gov record was submitted on 27 June 2025, first enrollment occurred on 15 July 2025, and the record was first publicly posted on 17 July 2025 (NCT07070661) [15]. Last enrollment was on 1 October 2025, and the final six-month assessment was completed on 30 April 2026. Outcomes are reported at the scheduled visits.

The study (NCT07070661) was approved by the Research Ethics Committee of the Antiguo Hospital Civil de Guadalajara “Fray Antonio Alcalde” (CEI 144/25) on 9 January 2025. Written informed consent was obtained from all participants. The study was conducted in accordance with the Declaration of Helsinki.

We selected the 2-month pain comparison after inspecting outcome trajectories; this was not prospectively specified. Accordingly, all inferential results are exploratory, and their p-values are not confirmatory hypothesis tests. Reporting follows CONSORT 2025 [16] and TIDieR [17].

### 2.2. Eligibility Criteria and Study Population

Eligibility was age 18–75 years, clinical CTS, and an abnormal median nerve electrodiagnostic study, without a Bland-grade restriction (NCT07070661). Exclusions were a normal median nerve study; severe thenar atrophy or muscle wasting; previous carpal tunnel surgery on the assessed limb; previous injection or shockwave/radial pressure wave therapy in the wrist or hand; cervical radiculopathy or peripheral polyneuropathy; systemic or musculoskeletal disease liable to interfere with assessment (including inflammatory arthritis, fibromyalgia, active infection, or cancer); systemic immunosuppressive treatment; and pregnancy or breastfeeding. All exclusions were applied before randomization, and any previous injection or shockwave/radial pressure wave therapy at those sites was exclusionary.

The enrolled population has mixed CTS severity, which limits the applicability of the findings to any specific severity grade. Bilateral CTS was allowed (nine participants: four constant, five ascending). The more symptomatic wrist was treated and assessed; the between-wrist NPRS difference ranged from 4 to 7 points. One wrist per participant was analyzed, pain and BCTQ answers referred to that wrist, and the other wrist was not treated.

### 2.3. Randomization and Blinding

We used simple randomization with a planned 1:1 ratio. From the first participant, the sequence was computer-generated in R 4.3.2 using randomizeR and concealed in sequentially numbered, opaque, sealed envelopes. An investigator who did not recruit participants or assess clinical outcomes prepared, held, and opened the envelopes, and each opening was recorded with the envelope number, the participant’s study code, and the assigned regimen. We did not use matching, minimization, blocking, or stratification. All 51 participants were randomized, 25 to the constant regimen and 26 to the ascending regimen.

The treating physician was not blinded and did not assess outcomes. Participants were not blinded, because the pressure and frequency differences were audible and palpable. Rehabilitation outcome assessors were intended to be blinded: they did not deliver treatment and were not told the allocation. We did not administer a blinding-success questionnaire, and we did not formally evaluate whether that blinding succeeded. The analyst was not blinded to the allocation.

### 2.4. Interventions and Concomitant Care

Each regimen consisted of three RPWT sessions, one per week, using a BTL-6000 radial device (BTL Industries Ltd., Stevenage, UK). The device is registered in Mexico as BTL-6000 RSWT, registration 1163E2023 SSA. A physician not involved in outcome assessment applied the transmitter perpendicular to the median nerve over the carpal tunnel using a fixed-point technique, with the participant seated, forearm supported and wrist slightly extended. Three-session courses have precedent in randomized CTS research [8]. The pressure, frequency, and impulse settings for both regimens are summarized in Table 1.

Both regimens delivered 2500 impulses per session and a nominal pressure–impulse product of 5000 bar–impulses. This index is not a measurement of acoustic energy delivered to tissue [4]. Theoretical emission duration was 250.0 s for the constant and 316.7 s for the ascending regimen, calculated from impulses divided by frequency and excluding pauses and setup. We did not measure clock time or acoustic energy: the exposure we recorded is the programmed impulse count, pressure, and frequency.

After the final session, a physician who did not assess outcomes taught each participant the same home program in person. Both arms received the printed leaflet and the accompanying audiovisual material. Participants were instructed to perform the program once daily on four to five days per week for four weeks, and the program was not progressed from week to week. Supplementary Methods S1 reproduces the exercises, hold times, and self-care advice. Our leaflet advised reassessment if numbness steadily worsened, if exercise increased pain, or if pain had not improved after four weeks. Our protocol specified this one program and we did not individualize it. Participants in both arms were telephoned to arrange appointments and to record each participant’s own statement of compliance with that instruction. Those calls were completed for the 24 constant and 19 ascending participants in the clinical analysis. The statement was not a count of completed home sessions, and we did not analyze it as an adherence rate. No interruptions or modifications of the home exercises were documented in those 24 constant and 19 ascending participants.

Our leaflet advised participants to avoid analgesics. We recorded NSAID use at follow-up and report it as observed; we did not code unknown or missing values as non-use (Table S1). The follow-up form asked about NSAID use, and we did not ask about splints, other analgesics, injections, or additional therapies.

### 2.5. Outcomes

#### Clinical Outcomes

Pain intensity was the protocol-designated main clinical outcome and was measured with the 11-point Numeric Pain Rating Scale (NPRS; 0, no pain; 10, the worst pain possible). Our protocol used the term visual analogue scale for this rating. At each assessment, it asked the participant to record current pain intensity. It did not define a longer recall period, and it did not ask for average pain or for worst pain over a stated interval. We did not recover a record of the question or the recall period that was used. The scale anchors are not interchangeable with the mean-pain and worst-pain change thresholds in Table S2. Our protocol designated no single primary assessment time. The secondary clinical outcomes retained in this report are the validated Spanish Boston Carpal Tunnel Questionnaire (BCTQ) symptom severity and functional status scales [18]. Pain and the BCTQ were assessed at baseline, at the third weekly session (post-treatment), and at 2, 4, and 6 months after the first treatment session. Grip strength is retained only as a baseline characteristic; longitudinal grip outcomes are excluded from this revised report.

The BCTQ symptom scale includes numbness and tingling, so patient-reported sensory symptoms were represented. We did not include systematic repeated quantitative sensory assessment, Tinel’s sign, or Phalen’s maneuver among the outcomes planned in our protocol.

### 2.6. Statistical Analysis

The original two-independent-means normal-approximation calculation assumed two-sided alpha 0.05, 80% power, a 2.0-point pain difference, and SD 2.1. The calculated 17.29 participants per arm became 21.61 after dividing by 0.80 for anticipated 20% attrition, rounded to 22 per arm (44 total). This was an attrition-inflated recruitment target, not a requirement for 44 analyzable participants. The original power assumption is reported unchanged; it does not establish power for the post hoc longitudinal comparison reported here. The non-confirmatory interpretation follows from the post hoc analysis and timepoint selection, post-randomization selection, and imprecision, rather than a comparison of the 43 analyzed participants with the recruitment target. No interim analysis was planned or conducted, and there was no formal stopping rule. We randomized 51 participants. The formula and future illustrative scenarios appear in Supplementary Methods S2 and Table S3.

The available clinical population comprised 43 participants (24 constant, 19 ascending). Seven randomized participants left the study (one constant, six ascending) and were not included. This population does not represent intention-to-treat analysis of all 51 randomized participants. Participants with baseline Bland grades above 3 were retained in the clinical analysis. A revision addendum dated 22 September 2026 specified the additional analyses before their execution; it remains post hoc relative to the study and known results.

The main exploratory comparison was the ascending-minus-constant difference in NPRS change from baseline to 2 months, estimated by REML using arm, categorical time, their interaction, and a participant-specific random intercept (compound-symmetric covariance). All scheduled visits are reported. Clinical estimates have unadjusted 95% confidence intervals; Holm-adjusted p-values and Bonferroni intervals across visits within outcome are supplementary. Sensitivity analyses used AR(1) and unstructured covariance. A further sensitivity analysis was restricted to the 27 participants aged 30–65 years with Bland grades 1–3; the composition of this subset is reported in Results. Two-month ANCOVA adjusted for baseline NPRS, with an additional model including age and baseline Bland > 3. ANCOVA intervals used conventional and HC3 standard errors.

Pooled-cohort models describe observed trajectories and do not estimate RPWT efficacy. In the conservative BCTQ analyses, we treated seven corrected cells as missing. In the main analysis, we retain those corrected means: each is the sum of the paper item scores divided by 11 or by 8. We report NSAID use as recorded; we did not code unknown or missing values as non-use. Existing imputation analyses concern missing measurements within the clinical cohort and cannot address the seven participants who left the study or the participant without clinical scores. The 2-month delta penalty does not supply that address: within these 43 participants no 2-month value is missing, so the penalty is inert and the unchanged estimates are not evidence about those exclusions. The additional procedures are in Supplementary Methods S2.

## 3. Results

### 3.1. Participants

**Figure 1:**
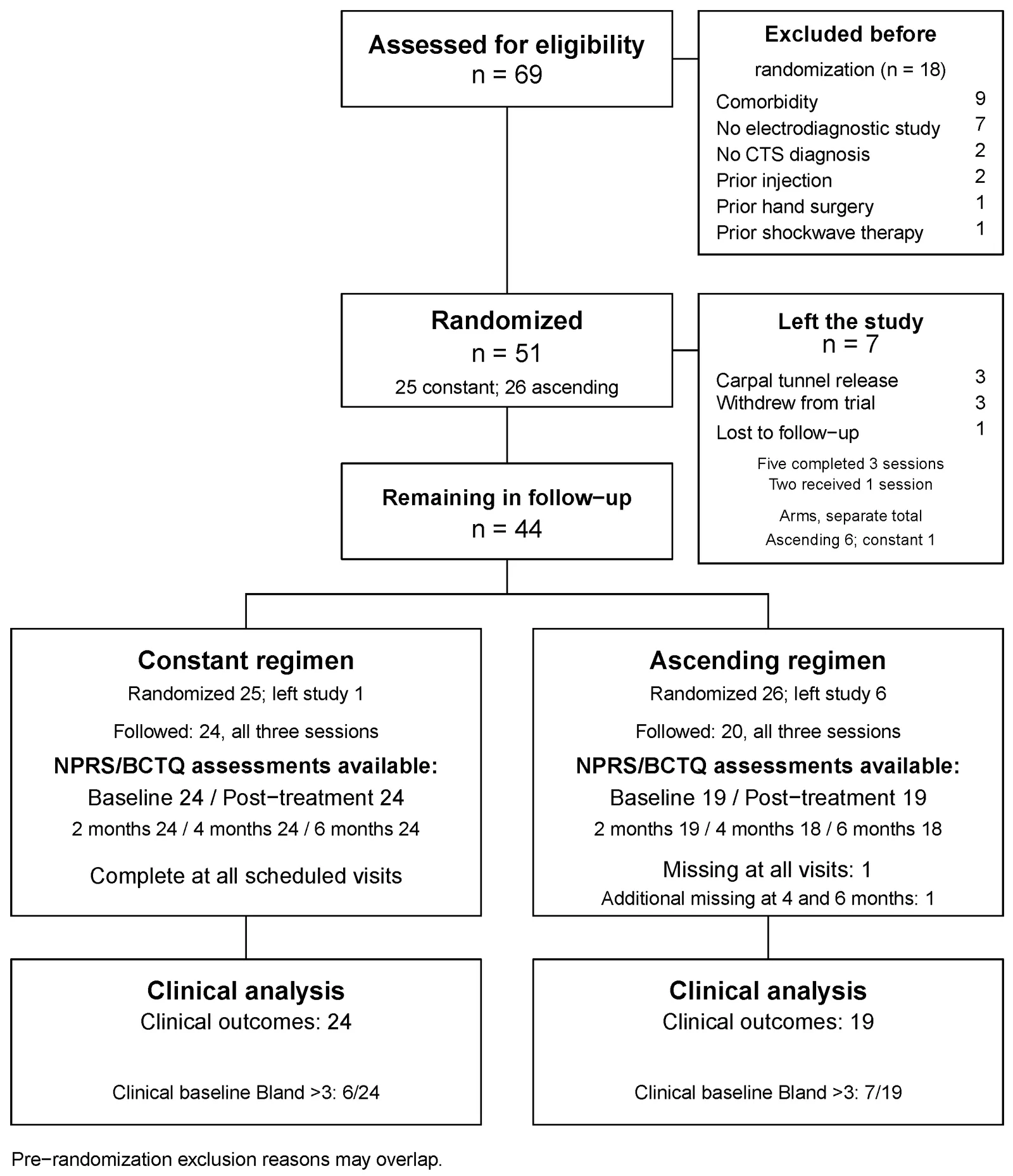
Participant flow and clinical analysis population. Seven participants left the study and were not included in the outcome populations. The reasons (three carpal tunnel releases, three withdrawals, and one loss to follow-up) and the arms (six ascending, one constant) are separate totals, not a cross-tabulation. Five of these seven had completed all three sessions before exclusion and two had received one session. Clinical outcomes were available for 43 of the 44 participants who remained in follow-up. The participant without clinical scores is not an eighth person who left. Scheduled-visit counts refer to NPRS/BCTQ, not treatment attendance.

Figure 1 summarizes participant flow and the clinical analysis population. Sixty-nine individuals were assessed and eighteen excluded before randomization. Reasons, which could overlap, were exclusionary comorbidity (9), no electrodiagnostic study (7), no CTS diagnosis (2), previous injection (2), previous hand surgery (1), and previous shockwave therapy (1). All 51 randomized participants (25 constant, 26 ascending) received at least one treatment session. Seven subsequently left the study and were not included in the outcome analyses. The recorded reasons were carpal tunnel release in three participants, withdrawal in three, and loss to follow-up in one. Separately, six of the seven had been assigned to the ascending regimen and one to the constant regimen. Five had completed all three sessions before exclusion, whereas two had received one session. Reasons for departure and allocation totals are reported as separate aggregates; a participant-level reason-by-arm cross-tabulation was not verified. Within two weeks of the participant’s first treatment session (per the logbook), treating physicians in the orthopedics department indicated carpal tunnel release for three of them, and those three left the protocol for that reason. The indication was part of the institution’s multidisciplinary care, outside the study protocol, and the study team did not control it.

These retention and imbalance patterns provide exploratory information about feasibility. Retention was 44/51 (86%) in follow-up and 43/51 (84%) for clinical scores, and attrition was asymmetric: six of the seven departures occurred in the ascending arm. Separately, three participants were indicated for carpal tunnel release within two weeks of the first session. Because a verified reason-by-arm cross-tabulation is unavailable, those indications cannot be assigned to a regimen. Material baseline imbalance was observed in this small analyzed cohort: grip strength SMD 0.624 (20.2 versus 25.5 kg), Bland grade SMD 0.356 (2.6 versus 3.1), and symptom duration SMD 0.312 (28.7 versus 39.2 months). These observations do not establish that simple randomization is inherently inadequate. Post hoc covariate adjustment does not eliminate uncertainty or resolve selection after randomization.

Forty-four participants remained in follow-up (24 constant, 20 ascending), and all completed three sessions. One ascending-arm participant remained in follow-up but had no clinical outcome scores in the analytical dataset, leaving 43 for clinical analysis. This participant was not an additional departure: seven participants left the study, whereas eight randomized participants were absent from the clinical analysis. These counts therefore refer to distinct populations. NPRS and BCTQ were available for all 43 clinical participants at baseline, post-treatment, and 2 months; within this population, one ascending-arm participant had missing values at 4 and 6 months.

All 44 participants followed for outcomes were aged 32–74 years. Among the 43 in the clinical analysis, 13 had a baseline Bland grade above 3 (6/24 constant, 7/19 ascending): nine grade 4 and four grade 5. A sensitivity subset restricted to age 30–65 years and Bland grades 1–3 comprised 27 participants (17 constant, 10 ascending). Table 2 describes the clinical population; Supplementary Tables compare age and Bland grade with that narrower window and give the categorical Bland distribution. Close NPRS and BCTQ symptom means coexisted with larger differences in grip strength, functional status, and Bland grade.

### 3.2. Main Exploratory Between-Regimen Comparison

**Figure 2:**
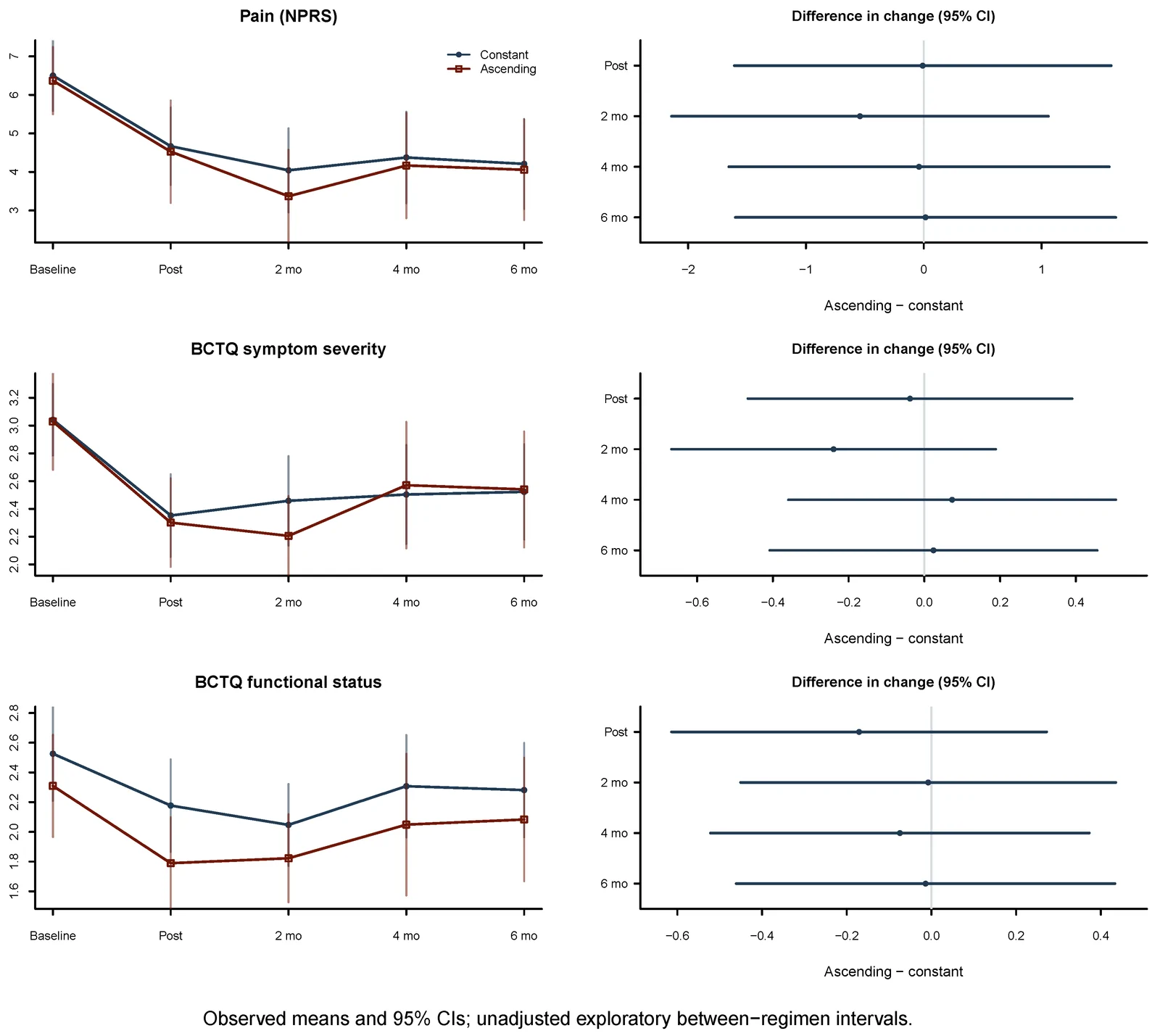
Observed NPRS and BCTQ trajectories and exploratory between-regimen differences in change. (**Left**): observed means and 95% confidence intervals. (**Right**): ascending minus constant contrasts with unadjusted 95% intervals from the random-intercept model. NPRS and BCTQ clinical population: 24 constant and 19 ascending, with one ascending participant missing at 4 and 6 months. Post-treatment is the third weekly session, and the 2-, 4-, and 6-month visits are timed from the first treatment session. These contrasts were not prospectively selected confirmatory tests.

The difference in NPRS change at 2 months was −0.54 points (95% CI −2.14 to 1.06; ascending minus constant). Figure 2 shows the NPRS and BCTQ trajectories and contrasts. Table 3 shows the unadjusted pain and BCTQ contrasts. Multiplicity-adjusted NPRS and BCTQ contrasts are in Table S4. These estimates are inconclusive about superiority and do not establish equivalence or non-inferiority. The imprecise interval is partly a consequence of the small realized sample and the baseline imbalance described in Section 3.1. The selected timepoint and p-values must be interpreted within the post hoc framework.

### 3.3. Descriptive Cohort Trajectories

The pooled estimated NPRS change at 2 months was −2.70 points (simultaneous 95% CI −3.78 to −1.62). Pooled changes and individual-response benchmarks are reported in Tables S2 and S6. They describe observed improvement in both arms during combined RPWT and exercise and cannot establish the contribution of either component. We did not measure home-session completion. Without a sham, exercise-only, or no-treatment comparator, this improvement cannot be attributed to RPWT rather than to exercise, natural history, or regression to the mean. Illustrative future sample-size calculations are confined to Table S3.

### 3.4. Sensitivity Analyses and Concomitant Medication

Alternative covariance models retained the 2-month pain estimate of −0.54 with wider intervals. In the subset aged 30–65 years with Bland grades 1–3, the estimate was −0.94 (random-intercept 95% CI −2.92 to 1.05). Baseline-pain ANCOVA gave −0.66 (HC3 95% CI −2.33 to 1.01); additionally adjusting for age and Bland >3 gave −0.66 (−2.47 to 1.16). Conventional and HC3 results are presented together in Table S5. These sensitivities do not resolve post-randomization selection.

The conservative BCTQ between-regimen analyses were estimable and their intervals included zero at all visits (Table S7). Those analyses omit the seven corrected cells; the main analysis retains them. Table S8 retains the limited within-cohort missing-data sensitivity; there were no missing 2-month clinical scores among the 43 analyzed participants. Descriptive pooled-cohort sensitivity analyses and covariance selection are reported in Tables S9 and S10, respectively.

Recorded NSAID use in the constant arm was 2/24 post-treatment, 1/24 at 2 months, 2/24 at 4 months, and 1/24 at 6 months. Corresponding ascending-arm counts were 1/19, 1/19, 1/18, and 1/18. We did not record NSAID use at baseline; one ascending participant had unknown use at each late visit. We report these observations and the unknown values separately in Table S1.

### 3.5. Adverse Events

We actively assessed adverse events during treatment and follow-up: the treating physician examined the treated wrist at the end of each of the three weekly sessions, and the study nurse asked about new symptoms at the treated wrist at the scheduled telephone contacts; findings were recorded in the study logbook. No dedicated adverse-event form or standardized severity scale was used. In the followed population, each arm had four participants with pain and two with erythema; the erythema cases overlapped the pain cases, giving four unique affected participants per arm (4/24 constant; 4/20 ascending). The pain was mild and the erythema was associated with the procedure. Both were transient, and we gave no treatment for them. No deaths or serious adverse events were reported among the 44 participants with available safety follow-up. The seven who left had also been exposed: five had completed all three sessions before exclusion and two had received one session, so this statement does not evaluate safety for every treated participant. Three of the seven who left the study underwent carpal tunnel release. The arms of the seven are a separate total. Within two weeks of the participant’s first treatment session (per the logbook), treating physicians in the orthopedics department indicated those releases, and the participants left the protocol for that reason. The indication was part of the institution’s multidisciplinary care, outside the study protocol, and the study team did not control it. The three carpal tunnel releases are reported as intercurrent clinical events; causality and seriousness were not formally adjudicated, and these data neither establish nor exclude a relationship with radial pressure wave therapy. We report the timing of the indication, not a date of operation. We do not assign these three releases to an arm, and we do not split the session counts by this reason. This report includes no safety assessment after they left the protocol.

## 4. Discussion

The descriptive information on retention, outcome availability, and tolerability identifies considerations for future trial planning. Follow-up retention was 44/51 (86%), and clinical scores were available for 43/51 (84%). Mild, transient pain and erythema were recorded among participants who remained in follow-up; safety information was incomplete for those who left. Because no prospective feasibility success criteria were defined, these observations do not establish feasibility. The observed SD of pain change (3.11 points) is the variability used in the illustrative planning scenarios in Table S3. It differs from the cohort baseline SD (1.99 points) and the original planning assumption (2.1 points); these scenarios do not retrospectively justify the completed sample. The main exploratory comparison estimated a small and imprecise difference in pain change at 2 months (−0.54 NPRS points; 95% CI −2.14 to 1.06). Our analysis plan followed data finalization and the timepoint followed trajectory inspection, which precludes confirmatory interpretation; data-dependent selection of the assessment time can inflate type I error. Baseline adjustment, alternative covariance structures, and restriction to age 30–65 years and Bland grades 1–3 retained intervals including zero. These findings do not establish superiority, equivalence, or non-inferiority.

The interventions were complete regimens differing simultaneously in pressure, frequency, and theoretical emission duration. Their comparison cannot isolate sequencing or attribute a difference specifically to modulation. The constant regimen had shorter theoretical emission time, but equal impulse counts and nominal pressure–impulse products do not measure equivalent acoustic energy or establish that it was therapeutically underdosed. The shorter emission time may have influenced outcomes, including by attenuating any effect of the constant regimen, but the available measurements cannot establish the direction or magnitude of such an influence. No statistical adjustment can separate variables that were fixed together by assigned regimen. Three-session courses have trial precedent [8], while the optimal number of sessions remains uncertain; a longer course could yield different outcomes.

Attrition and baseline imbalance are core pilot findings, not merely limitations. Seven participants left the analyzed populations after randomization (one constant, six ascending); five of the seven had completed all three sessions before exclusion and two had received one session. Within two weeks of the participant’s first treatment session (per the logbook), the orthopedics department indicated carpal tunnel release for three of them, and they left the protocol for that reason; the indication was part of the institution’s multidisciplinary care, outside the study protocol, and the study team did not control it. Three withdrew and one was lost to follow-up. The reasons and the arm counts are separate totals, not a cross-tabulation, and their exclusion precludes a classic intention-to-treat analysis. For a pilot, these patterns identify issues to address prospectively: (i) the observed baseline imbalance supports considering block or stratified randomization on baseline severity and, if appropriate, grip strength in a future trial, without establishing that simple randomization is inherently inadequate; (ii) early surgical indications warrant prospective monitoring of intercurrent events, but the unavailable reason-by-arm cross-tabulation precludes assigning those indications to either regimen; and (iii) post hoc adjustment may account for measured baseline covariates but does not eliminate uncertainty or repair post-randomization selection. Baseline resemblance in two measures does not verify allocation integrity, and the observed split of 25 constant and 26 ascending participants does not by itself verify that the concealed sequence was followed.

Thirteen of the forty-three clinical participants had a baseline Bland grade above 3, proportionally more frequent in the ascending arm. The between-regimen estimate may partly reflect baseline severity; adjustment for age and Bland grade above 3 gave −0.66 points but cannot exclude residual confounding. The enrolled sample is therefore a mixed-severity population, and applicability to any specific severity grade is limited; the 27-participant subset restricted to age 30–65 years and Bland grades 1–3 is a sensitivity analysis, not the enrolled population.

Mean pain improved in both arms. Pooled improvement cannot establish RPWT efficacy. Both groups received RPWT, home exercises, and associated self-care advice, without a sham, exercise-only, or no-treatment comparator. Device effects, exercise, expectations, natural history, and regression to the mean cannot be separated. Improvement in comparator groups or untreated CTS has been reported elsewhere [19,20,21,22], but those studies do not supply a control for this cohort. We did not measure home-session completion: we issued the same leaflet to both arms, and the exposure we logged is attendance at the clinic sessions.

Conventional Bland grading and contemporary predictive models answer different questions. The studies by Inui et al. and Sahin et al. illustrate prediction of postoperative outcomes and severity classification from richer datasets [11,12]. They contextualize severity assessment but do not validate either RPWT regimen. Our dataset is too small to support development and reliable validation of a new machine-learning model.

The remaining limitations include imprecision, post hoc analysis and timepoint selection, seven participants who left the study (one constant, six ascending), baseline Bland grades above 3, participant and therapist unblinding, and the absence of a sham or exercise-only comparator. We did not administer a blinding-success questionnaire, and our protocol did not include serial sensory examination or provocative signs as outcomes. Electrodiagnostic outcome analyses have been excluded from this report because of selection and standardization limitations; no neurophysiological treatment effects are inferred. These limitations cannot be resolved through additional modeling and prevent a clinical recommendation favoring either regimen.

## 5. Conclusions

This pilot exploratory comparison provides descriptive information on clinical trajectories and methodological considerations for future trial design. These observations do not establish feasibility: no prospective success criteria were specified, and follow-up was incomplete for participants who left. Retention was 44/51 (86%) in follow-up and 43/51 (84%) for clinical scores; attrition was asymmetric (six of seven departures in the ascending arm), and material baseline imbalance was observed (grip strength SMD 0.624; Bland grade SMD 0.356; symptom duration SMD 0.312). The main exploratory comparison found a small, imprecise between-regimen difference in pain change at 2 months (−0.54 NPRS points; 95% CI −2.14 to 1.06). Mean pain improved in both arms, but because both groups received exercises, home-session completion was not measured, and no sham or exercise-only control was included, this improvement cannot be causally attributed to the RPWT device. Because pressure, frequency, and theoretical emission duration differed jointly, the comparison concerns complete regimens and cannot isolate an effect of modulation or sequencing. The exploratory findings do not support recommending one regimen over the other. They may inform the design of a future definitive trial incorporating stratified or block randomization, a sham or exercise-only comparator, and sample-size planning informed by the observed variability.

## Figures and Tables

**Table 1:** RPWT regimens. Each arm received three weekly sessions.

Regimen/Phase	Impulses	Pressure (bar)	Frequency (Hz)	Theoretical Emission (s)
Constant	2500	2.0	10	250.0
Ascending: phase 1	800	1.5	5	160.0
Ascending: phase 2	900	2.0	10	90.0
Ascending: phase 3	800	2.5	12	66.7
Ascending: total	2500	—	—	316.7

The ascending phases occurred consecutively within each session. Durations are calculated, exclude pauses/setup, and are not observed session durations. The nominal pressure–impulse product is 5000 bar–impulses in both arms.

**Table 2:** Baseline characteristics of the cohort and of each arm. Standardized mean differences (SMD) are reported; following CONSORT 2025, no baseline significance testing is performed.

Variable	Cohort (*n* = 43)	Constant (*n* = 24)	Ascending (*n* = 19)	SMD
Age (years)	49.91 ± 10.34	51.08 ± 9.05	48.42 ± 11.86	−0.252
BMI (kg/m^2^)	27.93 ± 5.15	27.70 ± 4.99	28.23 ± 5.47	0.100
Symptom duration (months)	33.30 ± 33.03	28.67 ± 28.28	39.16 ± 38.19	0.312
Baseline Bland grade	2.81 ± 1.22	2.62 ± 1.31	3.05 ± 1.08	0.356
Baseline grip strength (kg)	22.53 ± 8.89	20.21 ± 9.55	25.47 ± 7.18	0.624
Baseline NPRS	6.44 ± 1.99	6.50 ± 2.17	6.37 ± 1.80	−0.066
Baseline BCTQ-SS	3.04 ± 0.65	3.04 ± 0.61	3.03 ± 0.72	−0.019
Baseline BCTQ-FS	2.43 ± 0.73	2.53 ± 0.75	2.31 ± 0.71	−0.296
Female sex, n (%)	38 (88.4%)	22/24	16/19	—
Right side, n (%)	34 (79.1%)	18/24	16/19	—

Values are mean ± standard deviation unless otherwise stated; the cohort column has *n* = 43 throughout. SMD computed as (ascending − constant)/pooled standard deviation. The largest absolute SMDs are for grip strength (0.624), Bland grade (0.356), and symptom duration (0.312); see the pilot-findings paragraph of Section 3.1. Abbreviations: BCTQ-FS, Boston Carpal Tunnel Questionnaire Functional Status; BCTQ-SS, Boston Carpal Tunnel Questionnaire Symptom Severity; BMI, body mass index; NPRS, numeric pain rating scale; SMD, standardized mean difference.

**Table 3:** Exploratory between-regimen comparisons. Between-arm difference in change from baseline (ascending − constant); a negative value favors the ascending arm. Estimates are unadjusted for multiplicity, consistent with the exploratory nature of the trial.

Outcome	Visit	Difference	95% CI	*p*
Pain (NPRS, 0–10)	Post-treatment	−0.01	−1.61 to 1.59	0.991
	2 months	−0.54	−2.14 to 1.06	0.505
	4 months	−0.04	−1.66 to 1.57	0.961
	6 months	0.02	−1.60 to 1.63	0.985
BCTQ–Symptom Severity (1–5)	Post-treatment	−0.04	−0.47 to 0.39	0.861
	2 months	−0.24	−0.67 to 0.19	0.271
	4 months	0.07	−0.36 to 0.51	0.738
	6 months	0.02	−0.41 to 0.46	0.912
BCTQ–Functional Status (1–5)	Post-treatment	−0.17	−0.61 to 0.27	0.448
	2 months	−0.01	−0.45 to 0.44	0.973
	4 months	−0.07	−0.52 to 0.37	0.742
	6 months	−0.01	−0.46 to 0.43	0.951
**Arm × time interaction, by covariance structure **				
Pain (NPRS, 0–10)	compound symmetry *p* = 0.952; AR(1) *p* = 0.893; unstructured *p* = 0.860			
BCTQ–Symptom Severity (1–5)	compound symmetry *p* = 0.653; AR(1) *p* = 0.471; unstructured *p* = 0.278			
BCTQ–Functional Status (1–5)	compound symmetry *p* = 0.935; AR(1) *p* = 0.849; unstructured *p* = 0.813			

REML model with a participant-specific random intercept and homogeneous residual variance (compound symmetry). Covariance alternatives and the sensitivity restricted to age 30–65 years and Bland grades 1–3 for the main exploratory pain contrast are in Table S5. Intervals include zero; they do not establish equivalence. Abbreviations: AR(1), first-order autoregressive; CI, confidence interval; BCTQ-FS, Boston Carpal Tunnel Questionnaire Functional Status; BCTQ-SS, Boston Carpal Tunnel Questionnaire Symptom Severity; NPRS, numeric pain rating scale.

## Data Availability

The de-identified analytic dataset and R code are publicly available in Zenodo, version 1.0, under CC BY 4.0: https://doi.org/10.5281/zenodo.23076030.

## Supplementary Materials

The following supporting information can be downloaded at https://www.mdpi.com/article/10.3390/jcm15218209/s1: Supplementary Methods S1: Home exercise programme; Supplementary Methods S2: Statistical detail; Supplementary Methods S3: Targeted source reconciliation; Table S1: Recorded NSAID use; Table S2: Clinically important change thresholds used, and responder proportions by criterion and visit; Table S3: Illustrative sample-size scenarios based on observed variability; Table S4: Between-arm difference in change from baseline (ascending minus constant), with Holm adjustment for multiplicity. Complements Table 3 of the manuscript, which reports the unadjusted pain and BCTQ estimates; Table S5: Exploratory sensitivity analyses of the between-regimen pain comparison at 2 months; Table S6: Descriptive analysis. Trajectory of the whole cohort (both arms pooled, with arm as an adjustment covariate) and responder proportions; Table S7: Conservative BCTQ sensitivity: corrected cells treated as missing; Table S8: Pooled-cohort pain change under multiple imputation and a pattern-mixture delta penalty applied to imputed values in the ascending arm; Table S9: Sensitivity analyses of pooled-cohort change from baseline to 2 months; Table S10: Covariance selection by AIC for the pooled-cohort models; File S1: CONSORT 2025 reporting checklist; File S2: TIDieR intervention checklist.

## Abbreviations

BCTQ	Boston Carpal Tunnel Questionnaire
CI	Confidence interval
CTS	Carpal tunnel syndrome
NPRS	Numeric Pain Rating Scale
RPWT	Radial pressure wave therapy
SD	Standard deviation
