# Supplementary material for: Constant Versus Ascending Radial Pressure Wave Therapy for Pain Management in Carpal Tunnel Syndrome: A Pilot Exploratory Study
Source: J Clin Med. 2026 Oct 22;15(21):8209. doi: 10.3390/jcm15218209 (PMC13764262; doi:10.3390/jcm15218209)
Supplement: Supplementary file 1 [file jcm-15-08209-s001.zip › jcm-4585538-supplementary.pdf]

Supplementary Materials: Constant Versus Ascending Radial Pressure Wave Therapy for Pain Management in Carpal Tunnel Syndrome: A Pilot Exploratory Study

Armando Tonatiuh Ávila-García [1,2], Ana Lilia Villagrana-Rodríguez [1,2], Elba Janette Ledezma-Miramontes [1], Emmanuel Mercado-Nuñez [1,2], Martha Alejandra García-Casillas [1,2], Erick José Ontiveros-Castellanos [1,2], Karen Chacón-Morales [1], Daniel Miller-Serrano [1], Diana Hazel Araiza-Quintana [1], Luis Fernando Herrera-Fuentes [3,4], Judith Carolina De Arcos-Jiménez [3,2] and Jaime Briseno-Ramirez [3,2,*]

Contents

Supplementary Methods S1–S3 document the home prescription, statistical details and targeted source reconciliation, respectively. Tables S1–S10 are numbered according to their first mention in the main manuscript:

- Table S1: recorded NSAID use, age and baseline Bland grade.
- Table S2: clinical response thresholds and sensitivity benchmarks.
- Table S3: illustrative sample-size scenarios for future trials.
- Table S4: multiplicity-adjusted between-regimen clinical contrasts.
- Table S5: direct between-regimen pain sensitivity analyses.
- Table S6: descriptive pooled-cohort trajectories and responder proportions.
- Table S7: conservative BCTQ analyses and corrected-cell documentation.
- Table S8: historical within-cohort missing-data sensitivity analyses.
- Table S9: descriptive pooled-cohort sensitivity analyses.
- Table S10: covariance selection for descriptive pooled-cohort models.

Files S1 and S2 are the CONSORT and TIDieR checklists, respectively.

Supplementary Methods S1. Home exercise programme

Both arms received the same home programme after the third RPWT session. A physician who did not assess outcomes taught it in person. Participants received a printed leaflet and the accompanying audiovisual material. Participants were instructed to perform the programme once daily on four to five days per week for four weeks, and the programme was not progressed from week to week. We did not individualize it. Participants in both arms were telephoned to arrange appointments and to record each participant's own statement of compliance with that instruction. Those calls were completed for the 24 constant and 19 ascending participants in the clinical analysis. The statement was not a count of completed home sessions, and we did not analyze it as an adherence rate. No interruptions or modifications of the home exercises were documented in those 24 constant and 19 ascending participants. We did not measure home-session completion. The exposure we recorded is attendance at the three clinic sessions. When both wrists were affected, the programme was taught for the more symptomatic wrist, the same wrist that received RPWT. Nine participants had bilateral involvement (4 constant, 5 ascending). On the 0–10 pain scale, the difference between the two wrists ranged from 4 to 7 points.

The leaflet instructed the following.

1. Apply a warm compress to the affected wrist for 10 minutes.
2. Actively flex and extend the wrist, with the elbow flexed to 90 degrees, for 10 repetitions.
3. Stretch the forearm extensors for 15 seconds, with the palm down and the fingers pointing down, and repeat five times.

4. Stretch the forearm flexors for 15 seconds, with the palm up and the wrist bent back, and repeat five times.
5. Hold each of the following positions for 3 seconds, in two sets of 5–10 repetitions: fingers and wrist straight; fingertips in a hook with the knuckles upward; a fist with the thumb outside the fingers; fingers extended with the thumb against the side of the hand; fingers straight, wrist back and thumb extended; and, in that position, a gentle thumb stretch with the other hand.
6. Apply gentle self-massage to the palm and wrist.
7. Slightly flex the fingers and press inward against light resistance, such as a soft elastic band or therapeutic putty.
8. Squeeze a soft therapy ball or a rolled towel, hold for 3 seconds and relax, for 10 repetitions.
9. A cold compress may be applied for 10 minutes if exercise causes pain.

Participants were advised to avoid significant pain during exercise and to seek reassessment if numbness steadily worsened, if the exercises increased pain, or if pain had not improved after four weeks. They were also advised to avoid sustained wrist flexion, repetitive or vibratory tasks and direct pressure on the heel of the hand, and to take an active pause every 30–45 minutes during hand or computer work.

Supplementary Methods S2. Statistical detail

The original sample calculation was $n = 2(1.96 + 0.84)^2(2.1)^2/(2.0)^2 = 17.2872$ per arm. Inflation for 20% attrition gave $17.2872/0.80 = 21.609$, rounded once at the final stage to 22 per arm. Pain was the 11-point numeric rating. Our protocol called this rating a visual analogue scale. The assumed difference is a planning parameter. The planning total was 44. There was no formal stopping rule, and 51 participants were randomized. Table S3 preserves illustrative future scenarios, not a new justification of the completed sample.

The revision addendum was dated before additional models were executed on 22 September 2026. All analyses remain post hoc. The 12 submitted clinical contrasts and confidence intervals were reproduced to numerical tolerance 10^{-7}. Main estimates retain the random-intercept REML model with arm, categorical time and interaction. Direct contrasts use the corresponding fixed-effect linear combinations; clinical intervals use model degrees of freedom. Pooled models have time and arm without interaction and are descriptive. Their historical covariance selection by AIC is retained in Table S10, separate from the fixed clinical reference model.

The 2-month ANCOVA models use baseline NPRS, then additionally age and baseline Bland >3. HC3 covariance uses squared residuals divided by $(1 - h_{ii})^2$ in the sandwich estimator, with residual-degree-of-freedom t intervals. Both conventional and HC3 estimates are reported. These additional ANCOVA specifications were listed in the post hoc revision addendum before fitting; they were not selected by statistical significance. No matching, minimization or assignment imputation was performed.

The revision script was run in R 4.6.1 and attached only the recommended package nlme 3.1-169. Linear models, distribution functions, Fisher exact tests and Wilcoxon tests used base R. For the random-intercept reference model, an interval uses the denominator degrees of freedom stored by nlme in `fixDF`, taking the smallest value among coefficients with nonzero weight in the contrast. For the clinical pain contrasts that denominator is 162. It equals 213 observed pain rows minus 43 participants minus the eight within-participant coefficients for time and the arm-by-time interaction. In the 27-participant subset the stored denominator is 100 for that model and 125 for the generalized least-squares fits. The AR(1) and unstructured fits are generalized least squares, and their intervals use

residual degrees of freedom, observed rows minus the number of fixed-effect coefficients: 203 in the clinical pain model. Both the conventional and HC3 ANCOVA intervals use the residual degrees of freedom of the linear model, 40 with baseline pain and 38 when age and baseline Bland >3 are added. These are the degrees of freedom already used for the reported intervals. They were not recalculated for this note.

Seven BCTQ cells had the subscale mean written in the cell intended for the item total. The next column divided that mean again by 11 (symptom severity) or 8 (functional status) and produced values below 1. The source questionnaires confirm the correction: the sum of the item scores divided by 11 or by 8 equals the mean used in the main analysis. These corrected means were already the values in the analyzed results. The second division was not analyzed. This revision checked that arithmetic; reproducing the submitted contrasts is compatible with the check, because those contrasts used the corrected means. These are seven subscale cells, not seven participants. A stable internal key (pseudonymous analytical code, visit and subscale) for the seven cells is preserved in the audit record so the omission pattern is reproducible from the analytical file; the key uses the same pseudonymous codes as the portable dataset and no direct identifiers. Conservative sensitivities set the same seven cells to missing, three symptom scores and four function scores. Table S7 lists each visit, the item sum, the corrected mean and the discarded second division. Our analytical file stores subscale means, not the individual item responses, so the item sums cannot be rebuilt from that file. The conservative between-regimen models and the pooled description are reported in Table S7. The pooled description uses the AR(1) covariance of the Table S9 reference, not the covariance selected in Table S10, and replaces the previous non-estimable entries. Table S6 reports the structure selected in Table S10.

We report NSAID use as recorded in Table S1. We did not code unknown or missing use as non-use. We do not treat a post-randomization cointervention automatically as a baseline confounder.

The clinical reference model does not impute. Rows with a missing outcome are omitted before fitting, and the fit is called with `na.action` set to `na.fail`, so a remaining missing outcome stops the fit rather than being filled in. A participant with any observed visit stays in the model. Within the 43-participant clinical analysis population, baseline and 2-month outcomes were observed for all participants; no values at these visits were imputed. This does not address post-randomization exclusions or eliminate the assumptions of the longitudinal model, which also uses the later visits and does not incorporate the eight participants without clinical scores. One ascending-arm participant is missing at 4 and 6 months, and those two rows are omitted. This handling is not the multiple-imputation or delta-penalty procedure in Table S8.

The historical within-cohort missing-data sensitivity in Table S8 concerns only 43 analyzed participants. Clinical data at 2 months are complete in that population, so every 2-month delta leaves the estimate unchanged. That invariance does not test post-randomization selection and is not evidence about the seven participants who left or about the participant without clinical scores: they are outside the calculation, and the penalty has no missing 2-month value on which to act. One participant lacks measurements at each late visit. These calculations stay within the 43 analyzed participants: they neither restore the seven participants who left the study nor create the additional participant's clinical scores. They are not a sensitivity analysis for all 51 randomized participants.

Table S6 retains the submitted paired Hedges' g. This revision did not recalculate it. For each outcome and visit, the change is the follow-up value minus the baseline value among complete pairs. With n pairs, mean change m and sample standard deviation s (divisor $n-1$), $g = Jm/s$, where $J = 1 - 3/(4(n-1)-1)$. A negative value is a decrease from baseline. Table S6 prints g to two decimal places. The calculation also stored a per-

centile bootstrap interval, 4000 resamples with seed 20260826, which Table S6 does not report. The 95% CI column of Table S6 is the Clopper–Pearson interval for the responder proportion, not an interval for g.

Supplementary Methods S3. Targeted source reconciliation

During revision, the source reconciliation was targeted: paired electrodiagnostic reports were recovered for one participant without clinical scores in the analytical dataset, one 4-month grade was corrected after source review, and seven BCTQ cells were reverified against the paper questionnaires. A full source-data audit of all 44 followed participants was not performed and is not claimed. The neurophysiological outcome analyses, figures and interpretations have been excluded from this report. Electrodiagnostic information is retained only for diagnostic confirmation, eligibility and baseline severity; no neurophysiological treatment effects are inferred. The BCTQ source checks and conservative clinical sensitivity analyses are described below.

Table S1. Recorded NSAID use.

Arm / visit	Clinical n	Use: yes	Use: no	Unknown
Constant / Baseline	24	0	0	24
Constant / Post-treatment	24	2	22	0
Constant / 2 months	24	1	23	0
Constant / 4 months	24	2	22	0
Constant / 6 months	24	1	23	0
Ascending / Baseline	19	0	0	19
Ascending / Post-treatment	19	1	18	0
Ascending / 2 months	19	1	18	0
Ascending / 4 months	19	1	17	1
Ascending / 6 months	19	1	17	1

We did not record NSAID use at baseline. We do not treat unknown or missing information as non-use. The denominator is the clinical analysis population. The follow-up form asked about NSAID use, and we did not ask about splints, other analgesics, injections or additional therapies.

Table S1 (continued). Age and Bland grade against a narrower window.

Arm	n	Age outside 30–65	Bland >3	Either	Inside both
Constant	24	1	6	7	17
Ascending	19	2	7	9	10

These columns compare the clinical population with age 30–65 years and Bland grades 1–3. That window is not the enrolment criterion. Enrolment used age 18–75 years and did not restrict Bland grade, so these counts are not eligibility deviations; grades above 3 describe a mixed-severity population and limit applicability to mild-to-moderate disease. If the enrolment criteria had excluded grades above 3, those counts would remain eligibility deviations, and calling the sample mixed severity would not remove them. All 44 participants followed for outcomes were aged 32–74 years. The three clinical participants outside 30–65 years were aged 72, 74 and 74, with Bland grades 2, 2 and 3. The 27 participants inside both limits are the unchanged sensitivity subset.

Table S1 (continued). Baseline Bland-grade distribution in the clinical population.

Grade	Constant (n=24)	Ascending (n=19)
1	5	2
2	8	3
3	5	7
4	3	6
5	3	1

These counts supplement the descriptive ordinal-grade summary in the baseline table.

Table S2. Clinically important change thresholds used, and responder proportions by criterion and visit.

Criterion and source	Post-treatment	2 m	4 m	6 m
**Pain (NPRS, 0–10)**				
NPRS reduction $\geq$ 1.5 points *(reference)*	53.5%	69.8%	59.5%	59.5%
GROC-anchored MCID in women with CTS, mean pain (PMID 37582474)				
NPRS reduction $\geq$ 2.5 points *(sensitivity)*	34.9%	55.8%	52.4%	52.4%
MCID for worst pain, same study (PMID 37582474)				
NPRS reduction $\geq$ 2.0 points *(sensitivity)*	53.5%	69.8%	59.5%	59.5%
Assumption of the protocol sample-size calculation (internal consistency)				
**BCTQ–Symptom Severity (1–5)**				
BCTQ-SS reduction $\geq$ 0.46 $\times$ baseline *(reference)*	11.6%	18.6%	9.5%	11.9%
Baseline-dependent MCID, AUC 0.88 (PMID 30463474)				
BCTQ-SS reduction $\geq$ 0.64 *(sensitivity)*	46.5%	53.5%	42.9%	35.7%
Fixed MCID in women with CTS (PMID 37582474)				
BCTQ-SS reduction $\geq$ 0.72 (MDC90) *(sensitivity)*	46.5%	53.5%	42.9%	35.7%
Minimal detectable change at 90% (PMID 32151499)				
**BCTQ–Functional Status (1–5)**				
BCTQ-FS reduction $\geq$ 0.28 $\times$ baseline *(reference)*	39.5%	37.2%	26.2%	26.2%
Baseline-dependent MCID, AUC 0.84 (PMID 30463474)				
BCTQ-FS reduction $\geq$ 0.23 *(sensitivity)*	58.1%	65.1%	57.1%	52.4%
Fixed MCID in women with CTS (PMID 37582474)				
BCTQ-FS reduction $\geq$ 0.79 (MDC90) *(sensitivity)*	32.6%	30.2%	23.8%	26.2%
Minimal detectable change at 90% (PMID 32151499)				

Baseline-dependent thresholds are substantially more demanding than fixed ones, and that is their purpose: a participant starting with more symptoms must improve more to perceive a change. Exact Clopper–Pearson intervals for the reference thresholds (pain reduction $\geq$1.5 points, BCTQ-SS $\geq$0.46 $\times$ baseline, and BCTQ-FS $\geq$0.28 $\times$ baseline) are in Table S6. The sensitivity thresholds in this table do not have those intervals. The 1.5-point row is the mean-pain anchor, and the 2.5-point row is the worst-pain anchor, from the same external study (PMID 37582474). Those anchors are not interchangeable with each other. Neither is interchangeable with the 0–10 rating in this trial: the protocol asked for current pain intensity, it did not define a longer recall period, and we did not recover a record of the question or recall period that was used. The 2.0-point row is the sample-size planning assumption, not an anchor from that study. Abbreviations: AUC, area under the curve; CTS, carpal tunnel syndrome; GROC, global rating of change; MCID, minimal clinically important difference; MDC90, minimal detectable change at 90% confidence; BCTQ-FS, Boston Carpal Tunnel Questionnaire Functional Status; BCTQ-SS, Boston Carpal Tunnel Questionnaire Symptom Severity; NPRS, numeric pain rating scale.

Table S3. Illustrative sample-size scenarios based on observed variability.

Outcome	SD of change	Target difference	*n* per group	Total N allowing for 20% attrition
Pain (NPRS, 0–10)	3.11	1.00	152	380
		1.50	68	170
		2.00	38	95
BCTQ–Symptom Severity (1–5)	0.83	0.64	27	68
		0.72	21	53
BCTQ–Functional Status (1–5)	0.73	0.23	158	395
		0.79	14	35
**Precision achieved by the present trial (n=24 and 19)**				
Pain (NPRS, 0–10)	minimum detectable difference at 80% power: 2.67			
BCTQ–Symptom Severity (1–5)	minimum detectable difference at 80% power: 0.71			
BCTQ–Functional Status (1–5)	minimum detectable difference at 80% power: 0.63			

Two-sided alpha 0.05, power 80%. The standard deviation of change assumed in the original sample-size calculation was 2.10, against 3.11 observed, which multiplies the required sample size by 2.19. The last column is the total sample after allowing for 20% attrition, not the number per group. Those totals divide the uninflated total by 0.80. Targets are planning assumptions, not validated between-arm MCIDs; variance uncertainty and alternative designs are not incorporated. Abbreviations: SD, standard deviation; BCTQ-FS, Boston Carpal Tunnel Questionnaire Functional Status; BCTQ-SS, Boston Carpal Tunnel Questionnaire Symptom Severity; NPRS, numeric pain rating scale.

Table S4. Between-arm difference in change from baseline (ascending minus constant), with Holm adjustment for multiplicity. Complements Table 3 of the manuscript, which reports the unadjusted pain and BCTQ estimates.

Outcome	Visit	Difference	95% CI (Bonf.)	*p* (Holm)
Pain (NPRS, 0–10)	Post-treatment	−0.01	−2.05 to 2.04	1.000
	2 months	−0.54	−2.59 to 1.50	1.000
	4 months	−0.04	−2.11 to 2.03	1.000
	6 months	0.02	−2.05 to 2.08	1.000
BCTQ–Symptom Severity (1–5)	Post-treatment	−0.04	−0.59 to 0.51	1.000
	2 months	−0.24	−0.79 to 0.31	1.000
	4 months	0.07	−0.48 to 0.63	1.000
	6 months	0.02	−0.53 to 0.58	1.000
BCTQ–Functional Status (1–5)	Post-treatment	−0.17	−0.74 to 0.40	1.000
	2 months	−0.01	−0.57 to 0.56	1.000
	4 months	−0.07	−0.65 to 0.50	1.000
	6 months	−0.01	−0.59 to 0.56	1.000

Random-intercept model as in Table 3. Holm p-value adjustment and Bonferroni confidence intervals across four visits within each NPRS or BCTQ outcome. Negative values favour the ascending regimen for NPRS/BCTQ. Every adjusted interval includes zero.

Table S5. Exploratory sensitivity analyses of the between-regimen pain comparison at 2 months.

Population / covariance	C/A	A − C	95% CI	*p*
Clinical cohort / CS	24/19	−0.54	−2.14 to 1.06	0.505
Clinical cohort / AR1	24/19	−0.54	−2.26 to 1.17	0.534
Clinical cohort / UN	24/19	−0.54	−2.44 to 1.35	0.574
Draft-window subset / CS	17/10	−0.94	−2.92 to 1.05	0.353
Draft-window subset / AR1	17/10	−0.94	−3.17 to 1.30	0.410
Draft-window subset / UN	17/10	−0.94	−3.34 to 1.47	0.444

Difference in change from baseline, estimated with arm, categorical time and interaction by REML. AR(1) uses ordered visit indices; UN permits visit-specific variances. The narrower subset is age 30–65 years and Bland grades 1–3. It is not the enrolment criterion. We did not measure home-session completion, so the subset is not a fully adherent per-protocol population. The estimate was not recomputed.

Table S5 (continued). Revision ANCOVA sensitivities.

Covariates / variance	n	A − C	95% CI	*p*
Baseline pain / Conventional	43	−0.66	−2.26 to 0.93	0.407
Baseline pain / HC3	43	−0.66	−2.33 to 1.01	0.428
Baseline pain + age + severe Bland / Conventional	43	−0.66	−2.27 to 0.96	0.416
Baseline pain + age + severe Bland / HC3	43	−0.66	−2.47 to 1.16	0.469

Outcome: NPRS at 2 months. Both models adjust for baseline NPRS; the second adds age and baseline Bland >3. HC3 intervals use residual degrees of freedom. All 43 clinical participants have observed baseline and 2-month scores. These post hoc analyses cannot recover excluded participants or remove data-dependent timepoint selection.

Table S6. Descriptive analysis. Trajectory of the whole cohort (both arms pooled, with arm as an adjustment covariate) and responder proportions.

Outcome	Visit	Change (95% CI)	Hedges' g	Responders	95% CI
Pain (NPRS, 0–10)	Post-treatment	−1.84 (−2.69 to −0.98)	−0.77	23/43 (53.5%)	37.7 to 68.8
	2 months	−2.70 (−3.78 to −1.62)	−0.85	30/43 (69.8%)	53.9 to 82.8
	4 months	−2.11 (−3.31 to −0.91)	−0.74	25/42 (59.5%)	43.3 to 74.4
	6 months	−2.27 (−3.54 to −1.00)	−0.74	25/42 (59.5%)	43.3 to 74.4
BCTQ–Symptom Severity (1–5)	Post-treatment	−0.71 (−0.98 to −0.44)	−0.99	5/43 (11.6%)	3.9 to 25.1
	2 months	−0.69 (−1.01 to −0.37)	−0.82	8/43 (18.6%)	8.4 to 33.4
	4 months	−0.51 (−0.84 to −0.17)	−0.58	4/42 (9.5%)	2.7 to 22.6
	6 months	−0.51 (−0.84 to −0.17)	−0.58	5/42 (11.9%)	4.0 to 25.6
BCTQ–Functional Status (1–5)	Post-treatment	−0.42 (−0.67 to −0.18)	−0.62	17/43 (39.5%)	25.0 to 55.6
	2 months	−0.48 (−0.80 to −0.17)	−0.65	16/43 (37.2%)	23.0 to 53.3
	4 months	−0.25 (−0.60 to 0.10)	−0.30	11/42 (26.2%)	13.9 to 42.0
	6 months	−0.24 (−0.62 to 0.13)	−0.29	11/42 (26.2%)	13.9 to 42.0

Responder thresholds: pain ≥1.5 points; BCTQ symptoms ≥0.46 × baseline score; BCTQ function ≥0.28 × baseline score. Exact Clopper–Pearson intervals for responders. Model change intervals use Bonferroni adjustment across four visits within each NPRS or BCTQ outcome; p values use Holm. Individual thresholds are descriptive and do not establish a between-arm margin. Paired Hedges' g is defined in Supplementary Methods S2 and was not recalculated. The 95% CI column is the responder interval, not an interval for g. The change interval for each outcome uses the covariance selected in Table S10: AR(1) for pain and BCTQ–FS, and unstructured for BCTQ–SS. The 2-month BCTQ–SS interval therefore matches the unstructured row of Table S9 (−1.01 to −0.37), not the AR(1) reference interval (−0.99 to −0.39). Abbreviations: CI, confidence interval; BCTQ-FS, Boston Carpal Tunnel Questionnaire Functional Status; BCTQ-SS, Boston Carpal Tunnel Questionnaire Symptom Severity; NPRS, numeric pain rating scale.

Table S7. Conservative BCTQ sensitivity: corrected cells treated as missing.

Outcome / visit	Observations	A − C	95% CI	Holm p
BCTQ-SS / Post-treatment	210	−0.051	−0.484 to 0.381	1.000
BCTQ-SS / 2 months	210	−0.258	−0.690 to 0.174	0.961
BCTQ-SS / 4 months	210	0.037	−0.399 to 0.474	1.000
BCTQ-SS / 6 months	210	0.024	−0.409 to 0.458	1.000
BCTQ-FS / Post-treatment	209	−0.181	−0.629 to 0.268	1.000
BCTQ-FS / 2 months	209	−0.023	−0.471 to 0.426	1.000
BCTQ-FS / 4 months	209	−0.079	−0.532 to 0.373	1.000
BCTQ-FS / 6 months	209	−0.042	−0.495 to 0.410	1.000

The same random-intercept arm-by-time model as the clinical reference is used. Three symptom and four function cells are omitted. The Observations column counts every longitudinal row used in that model. For these 43 clinical participants that is five visits, minus two missing late scores, minus the three symptom or four function cells omitted above: 210 and 209. The same count is repeated on each row. It is not the number of participants and not the number observed at the visit named in that row. Estimates are between-regimen differences in change; intervals are unadjusted and p-values Holm-adjusted across four visits within outcome.

Table S7 (continued). Pooled AR(1) fits at 2 months.

Outcome	Observations	Pooled change	Simultaneous 95% CI	Holm p
BCTQ-SS	210	−0.682	−0.986 to −0.377	<0.001
BCTQ-FS	209	−0.474	−0.795 to −0.154	<0.001

The pooled description uses AR(1), the covariance of the Table S9 reference, after the seven cells are omitted. It does not use the unstructured covariance selected for BCTQ–SS in Table S10 and reported in Table S6. The observation count is that same total of longitudinal rows, not the number of participants. These results replace the previous non-estimable entries. The model remains post hoc and does not estimate efficacy.

Table S7 (continued). Seven corrected BCTQ cells.

Visit	Scale	Item sum	Items	Mean used	Divided again
6 months	Function	18	8	2.25	0.28125
Post-treatment	Symptoms	22	11	2.00	0.1818
Post-treatment	Function	14	8	1.75	0.21875
2 months	Symptoms	22	11	2.00	0.1818
2 months	Function	12	8	1.50	0.1875
4 months	Symptoms	18	11	1.636	0.1488
4 months	Function	16	8	2.00	0.2500

The mean used is the sum of the paper item scores divided by 11 (symptoms) or 8 (function). The spreadsheet had written that mean in the total cell, and the next column divided it again; that second result is not a BCTQ score and we did not analyze it. These corrected means were already the values in the analyzed results; this revision verified the arithmetic and did not replace them. Cell-level linkage is retained in the audit record using pseudonymous participant code, visit and subscale, as described in Supplementary Methods S2. The same seven cells are set to missing only in the sensitivity above. Our analytical file stores subscale means, not the individual item answers.

Table S8. Pooled-cohort pain change under multiple imputation and a pattern-mixture delta penalty applied to imputed values in the ascending arm.

Visit	Imputed values	Delta (SD)	Estimate	95% CI	*p*
2 months	0	0.00	−2.70	−3.63 to −1.77	1.2e-08
		0.50	−2.70	−3.63 to −1.77	1.2e-08
		1.00	−2.70	−3.63 to −1.77	1.2e-08
		1.50	−2.70	−3.63 to −1.77	1.2e-08
		2.00	−2.70	−3.63 to −1.77	1.2e-08
4 months	1	0.00	−2.10	−2.98 to −1.21	3.5e-06
		0.50	−2.06	−2.96 to −1.16	7.4e-06
		1.00	−2.03	−2.95 to −1.10	1.6e-05
		1.50	−1.99	−2.94 to −1.05	3.7e-05
		2.00	−1.96	−2.94 to −0.98	8.4e-05
6 months	1	0.00	−2.24	−3.18 to −1.29	3.6e-06
		0.50	−2.20	−3.16 to −1.24	7.5e-06
		1.00	−2.16	−3.15 to −1.18	1.7e-05
		1.50	−2.13	−3.14 to −1.12	3.7e-05
		2.00	−2.09	−3.13 to −1.05	8.2e-05

Twenty predictive-mean-matching imputations; pooled mean change and total variance combined using Rubin rules, with unadjusted normal-approximation intervals and p values. At the 2-month visit there are no missing values, so the delta penalty is inert by construction. Identical estimates across those 2-month rows are not evidence about the seven participants who left the study or about the participant without clinical scores. Those people are outside this table, and the penalty has no missing 2-month value on which to act. At 4 and 6 months there is a single imputed value. This analysis concerns pooled change within 43 participants; it neither restores the seven participants who left the study nor supplies the additional participant's clinical scores, and it does not estimate a between-arm treatment effect.

Table S9. Sensitivity analyses of pooled-cohort change from baseline to 2 months.

Analysis	*n*	Estimate	95% CI	*p*
**Pain (NPRS, 0–10)**				
Reference (AR(1))	43	−2.70	−3.78 to −1.62	7.5e-09
S1 Narrower window	27	−3.11	−4.49 to −1.73	3e-07
S2 Corrected BCTQ scores omitted	43	−2.70	−3.78 to −1.62	7.5e-09
S3 Targeted source-check participant omitted	42	−2.74	−3.84 to −1.64	8.2e-09
S4 Complete cases	42	−2.81	−3.90 to −1.72	2.7e-09
S6 Unstructured covariance	43	−2.70	−3.89 to −1.50	1.8e-07
S6 AR(1) covariance	43	−2.70	−3.78 to −1.62	7.5e-09
S6 Compound-symmetry covariance	43	−2.70	−3.70 to −1.69	<1e-09
**BCTQ–Symptom Severity (1–5)**				
Reference (AR(1))	43	−0.69	−0.99 to −0.39	1.1e-07
S1 Narrower window	27	−0.71	−1.08 to −0.34	1.1e-05
S2 Corrected BCTQ scores omitted	43	−0.68	−0.99 to −0.38	<0.001
S3 Targeted source-check participant omitted	42	−0.71	−1.02 to −0.41	6.4e-08
S4 Complete cases	42	−0.70	−1.01 to −0.39	1.2e-07
S6 Unstructured covariance	43	−0.69	−1.01 to −0.37	4e-07
S6 AR(1) covariance	43	−0.69	−0.99 to −0.39	1.1e-07
S6 Compound-symmetry covariance	43	−0.69	−0.96 to −0.42	2.6e-09
**BCTQ–Functional Status (1–5)**				
Reference (AR(1))	43	−0.48	−0.80 to −0.17	0.00048
S1 Narrower window	27	−0.47	−0.85 to −0.10	0.0054
S2 Corrected BCTQ scores omitted	43	−0.47	−0.79 to −0.15	<0.001
S3 Targeted source-check participant omitted	42	−0.51	−0.83 to −0.18	0.00031
S4 Complete cases	42	−0.48	−0.80 to −0.16	0.00067
S6 Unstructured covariance	43	−0.48	−0.76 to −0.20	8.9e-05
S6 AR(1) covariance	43	−0.48	−0.80 to −0.17	0.00048
S6 Compound-symmetry covariance	43	−0.48	−0.76 to −0.20	7.8e-05

Pooled descriptive changes only; these are not estimates of RPWT efficacy. Holm p-values and Bonferroni intervals across visits within outcome. S1: subset aged 30–65 years with Bland grades 1–3. This narrower window is not the enrolment criterion of age 18–75 years without a Bland-grade limit. S2: seven corrected BCTQ cells treated as missing; the pooled description is in Table S7. S3: historical clinical sensitivity omitting a participant identified during targeted source reconciliation; this does not imply its clinical measurements were invalid. S4: complete clinical cases. S6: covariance alternatives. The reference row is AR(1) for every outcome. Table S6 uses the covariance selected in Table S10; for BCTQ–SS that selection is unstructured (−1.01 to −0.37), not this AR(1) reference (−0.99 to −0.39). Table S7 uses this AR(1) reference after the seven cells are omitted. We did not code unknown or missing NSAID use as non-use; observed use is in Table S1.

Table S10. Covariance selection by AIC for the pooled-cohort models.

Outcome	Structure	AIC	BIC	Selected
Pain (NPRS, 0–10)	Unstructured	929.6	999.6	
	AR(1)	928.4	955.1	✓
	Compound symmetry	948.0	974.7	
BCTQ–Symptom Severity (1–5)	Unstructured	406.7	476.6	✓
	AR(1)	407.3	433.9	
	Compound symmetry	417.8	444.5	
BCTQ–Functional Status (1–5)	Unstructured	427.0	496.9	
	AR(1)	421.6	448.3	✓
	Compound symmetry	426.3	452.9	

Models here contain time and arm without an interaction and describe pooled change. They are distinct from the main random-intercept between-arm model; direct pain sensitivities appear in Table S5. Table S6 reports the structure selected here. Table S9 keeps an AR(1) reference for its sensitivities, including where this table selects unstructured for BCTQ–SS. The pooled rows of Table S7 use that AR(1) reference after the seven corrected cells are omitted; they do not use the selection in this table. Abbreviations: AIC, Akaike information criterion; AR(1), first-order autoregressive; BIC, Bayesian information criterion; BCTQ-FS, Boston Carpal Tunnel Questionnaire Functional Status; BCTQ-SS, Boston Carpal Tunnel Questionnaire Symptom Severity; NPRS, numeric pain rating scale.

File S1. CONSORT 2025 reporting checklist

Trial: NCT07070661. Constant versus ascending pressure–frequency RPWT regimens in carpal tunnel syndrome.

Item numbering follows the source guideline; topic labels are abbreviated. Responses describe the trial and identify the relevant manuscript sections. Unavailable methodological details are stated explicitly.

Source: Hopewell et al. CONSORT 2025 statement. *BMJ* 2025;389:e081123. Official CONSORT–SPIRIT checklist.

Item	Reporting topic	Trial information and location
1a	Randomized-trial identification	The title identifies a pilot exploratory study and names the constant and ascending regimens. Randomized allocation is stated in the abstract and Methods; the word randomized is not included in the reviewer-requested title.<br>*Location:* Title, p. 1
1b	Structured abstract	The abstract reports the design, interventions, analysis population, principal estimates and conclusions.<br>*Location:* Abstract, p. 1
2	Registration	ClinicalTrials.gov NCT07070661: first submitted 27 June 2025; first publicly posted 17 July 2025, after the investigator-reported first enrolment on 15 July. URL: https://clinicaltrials.gov/study/NCT07070661.<br>*Location:* Main 2.1, p. 3 (registration dates)
3	Protocol and analysis plan	Original protocol and post hoc SAP are distinguished from the revision addendum dated 22 September 2026. Supplementary Methods S1-S3 give the home programme, clinical statistical details and targeted source reconciliation. The addendum is described in Supplementary Methods S2 and is not part of the public Zenodo archive.<br>*Location:* Main 2.6, p. 5; Main Data Availability, p. 13
4	Data and code access	De-identified analytic data and R code are public in Zenodo, version 1.0, DOI 10.5281/zenodo.23076030 (CC BY 4.0).<br>*Location:* Main Data Availability, p. 13
5a	Funding and support	No external funding was received. There was therefore no external funder role to report.<br>*Location:* Main Funding, p. 12
5b	Competing interests	We declare no conflicts of interest.<br>*Location:* Main Conflicts of Interest, p. 13
6	Scientific rationale	The comparison concerns complete constant and ascending schedules with equal impulse counts and nominal pressure-impulse product. Pressure, frequency and theoretical emission duration differ jointly; sequencing is not isolated.<br>*Location:* Main 1, p. 2
7	Objectives	The revised report describes retention, available tolerability observations and baseline imbalance, and exploratory pain and BCTQ comparisons. The pilot exploratory framing was adopted retrospectively in Round 2; feasibility was not a prospectively specified primary objective. Future sample-size scenarios are in section 3.3 and Table S3.<br>*Location:* Main 1, p. 2; Main 3.3, p. 9
8	Patient/public involvement	None.<br>*Location:* Study records; not reported
9	Design and allocation ratio	Two-arm, parallel-group study with randomized allocation and a planned 1:1 ratio. The report was reframed retrospectively as a pilot exploratory study in Round 2.<br>*Location:* Main 2.1, pp. 2–3; Main 2.3, p. 3
10	Protocol and analysis changes	From the first participant, allocation used a sequence computer-generated in R 4.3.2 with randomizeR and concealed in sealed envelopes. The original protocol had named a manual draw, which we did not use. The planning total was 44; 51 participants were randomized, and there was no formal stopping rule. Pain was the 11-point numeric rating; our protocol called it a visual analogue scale. The home programme in Supplementary Methods S1 is the leaflet issued to both arms. The detailed SAP and 2-month comparison were post hoc. The enrolment criteria were age 18-75 years, as posted for NCT07070661, without a Bland-grade limit. A 27-participant sensitivity uses age 30-65 and Bland grades 1-3; that narrower window is not the enrolment criterion. Paired EDX reports were recovered for one participant whose clinical scores are not in the analytical dataset.<br>*Location:* Main 2.3, p. 3; Main 2.6, p. 5; Main 3.1, pp. 6–7
11	Setting	Department of Physical Medicine and Rehabilitation, Antiguo Hospital Civil de Guadalajara, Mexico.<br>*Location:* Main 2.1, pp. 2–3

Continued on next page

Item	Reporting topic	Trial information and location (continued)
12a	Participant eligibility	Adults aged 18-75 years, as posted for NCT07070661, with clinical CTS and an abnormal median-nerve electrodiagnostic study. The enrolment criteria do not restrict Bland grade. Grades above 3 were enrolled under those criteria; the sample is of mixed severity, and these grades are not eligibility deviations. If the criteria had excluded grades above 3, those enrolments would remain deviations. Exclusions included severe thenar atrophy or muscle wasting, previous injection in the wrist or hand and previous shockwave or radial pressure wave therapy in the wrist or hand. Any previous treatment of either kind was exclusionary and was applied before randomization. Bilateral CTS was not excluded. Nine participants had bilateral involvement (4 constant, 5 ascending); the more symptomatic wrist was treated and assessed, and the between-wrist difference on the 0-10 pain scale ranged from 4 to 7 points. One wrist was analyzed per participant. All 44 participants followed for outcomes were aged 32-74 years. Baseline Bland grades above 3 are reported.<br>*Location:* Main 2.2, p. 3; Main 3.1, pp. 6–7
12b	Site/provider eligibility	Single-centre trial in the hospital rehabilitation service. RPWT was delivered by a physician in that service who did not assess outcomes.<br>*Location:* Main 2.1, pp. 2–3; Main 2.4, pp. 3–4
13	Intervention and comparator	Constant and ascending RPWT parameters, nominal dose, sessions, position, application technique, device and the common home exercise programme are described. File S2 provides the item-level TIDieR mapping.<br>*Location:* Main 2.4, pp. 3–4
14	Outcomes and time points	Pain and BCTQ: baseline, the third weekly session (post-treatment), and 2, 4 and 6 months after the first treatment session. Grip strength is retained only as a baseline characteristic; longitudinal grip outcomes are excluded from this report. NPRS was the main clinical outcome. The protocol asked for current pain intensity at each assessment. It did not define a longer recall period and did not ask for average pain or worst pain over an interval. We did not recover a record of the question or recall period that was used. The 1.5-point and 2.5-point anchors in Table S2 are mean-pain and worst-pain thresholds from another study and are not interchangeable with each other or with this rating. No single timepoint was prospectively designated. We selected the 2-month comparison after inspecting trajectories. BCTQ includes sensory symptoms; we did not plan systematic repeated sensory examination or provocative signs as outcomes.<br>*Location:* Main 2.5, pp. 4–5; Main 2.6, p. 5
15	Harms assessment	Adverse events were actively assessed: the treating physician examined the treated wrist at the end of each of the three weekly sessions, and the study nurse asked about new symptoms at the treated wrist at the scheduled telephone contacts; findings were recorded in the study logbook. No dedicated adverse-event form or standardized severity scale was used. Recorded events were pain or erythema; counts and overlap are reported. The leaflet instructed reassessment if exercises increased pain or numbness.<br>*Location:* Main 3.5, p. 10
16a	Sample-size assumptions	Original two-independent-means normal approximation: two-sided alpha .05, power .80, assumed pain difference 2, SD 2.1, 20% attrition; 17.2872/.80 rounded to 22/arm. The 44-participant total was an attrition-inflated recruitment target, not a requirement for 44 analyzable participants. These original assumptions do not establish power for the post hoc longitudinal comparison. Table S3 contains illustrative future scenarios.<br>*Location:* Main 2.6, p. 5; Main 3.3, p. 9
16b	Interim/stopping rules	No interim analysis was planned or conducted, and there was no formal stopping rule. The planning total was 44 participants; 51 were randomized.<br>*Location:* Main 2.6, p. 5
17a	Sequence generation	The allocation sequence was computer-generated in R 4.3.2 with randomizeR from the first participant. The original protocol had named a manual draw, which was not used.<br>*Location:* Main 2.3, p. 3
17b	Randomization type	Simple randomization with a 1:1 ratio. Matching, minimization, blocking and stratification were not used.<br>*Location:* Main 2.3, p. 3
18	Concealment mechanism	Sequentially numbered, opaque, sealed envelopes (SNOSE), prepared, held and opened by an investigator independent of recruitment and clinical assessment.<br>*Location:* Main 2.3, p. 3
19	Allocation implementation	Envelopes were prepared, held and opened by the investigator who did not recruit participants or assess outcomes.<br>*Location:* Main 2.3, p. 3
20a	Who was blinded	Participants and the treating physician were not blinded. Rehabilitation outcome assessors were intended to be blinded. The analyst was not blinded. Blinding success was not formally evaluated.<br>*Location:* Main 2.3, p. 3
20b	Blinding procedure	Pressure and frequency differences were audible and palpable, so participants could not be blinded. The treating physician delivered treatment and did not assess outcomes. Outcome assessors did not deliver treatment and were not told the allocation. We did not administer a blinding-success questionnaire.<br>*Location:* Main 2.3, p. 3
21a	Analysis methods	Main exploratory arm-by-time random-intercept REML comparison, with AR(1), UN and a sensitivity restricted to age 30-65 years and Bland grades 1-3. Revision ANCOVA adjusts for baseline pain, then age and baseline Bland >3, with conventional and HC3 intervals. The revision script used R 4.6.1 and nlme 3.1-169. Random-intercept intervals use the nlme denominator degrees of freedom of the contrast; generalized least squares and ANCOVA intervals use residual degrees of freedom. All inference remains post hoc.<br>*Location:* Main 2.6, p. 5

Continued on next page

Item	Reporting topic	Trial information and location (continued)
21b	Analysis population	Clinical population 43 (24 constant/19 ascending); Randomized totals were 25 constant and 26 ascending. Seven participants left the study (one constant, six ascending). This is an available clinical population, not an intention-to-treat analysis of all 51 randomized participants. *Location:* Main 2.6, p. 5; Main 3.1, pp. 6–7
21c	Missing measurements	Within-cohort models and imputation do not address the seven participants who left the study or the participant without clinical scores. The clinical reference model omits rows with a missing outcome and does not impute. Pain and the BCTQ are complete for all 43 at 2 months. The 2-month delta penalty in Table S8 is inert for that reason, and the unchanged estimates are not evidence about the seven who left. *Location:* Main 2.6, p. 5
21d	Additional analyses	Additional baseline-adjusted pain analyses and conservative BCTQ models are explicitly post hoc. The seven corrected BCTQ means equal the paper item sums divided by 11 or 8 and are listed in Table S7; the sensitivity treats those cells as missing. The pooled BCTQ description in Table S7 replaces the previous non-estimable entries. We report NSAID use as recorded, and we retained unknown values. *Location:* Main 2.6, p. 5; Main 3.4, pp. 9–10
22a	Participant flow	Figure 1 reports 69 assessed, 18 excluded before randomization, 51 randomized (25 constant, 26 ascending), 44 remaining in follow-up and 43 with clinical scores. Seven left the study (six ascending, one constant); the additional participant without clinical scores is not an eighth departure. *Location:* Main 3.1, pp. 6–7
22b	Losses and exclusions	The seven who left are reported as two separate totals, not a reason-by-arm cross-tabulation: six ascending and one constant; three carpal tunnel releases, three withdrawals and one loss to follow-up. Five of the seven had completed all three sessions before exclusion and two had received one session. Those session counts are not split by reason or by arm. Within two weeks of the participant's first treatment session (per the logbook) the orthopedics department indicated release for three of them, and they left the protocol for that reason. That indication was part of the institution's multidisciplinary care, outside the study protocol, and the study team did not control it. A separate ascending-arm participant has no clinical scores in the dataset and is not an eighth person who left. Seven left; eight are absent from the clinical analysis. One other ascending-arm participant has missing clinical values at 4 and 6 months. *Location:* Main 3.1, pp. 6–7; Main 3.5, p. 10
23a	Recruitment/follow-up dates	Recruitment took place from 15 July to 1 October 2025. Scheduled visits were timed from the first treatment session: post-treatment was the third weekly session, and the 2-, 4- and 6-month visits were counted from the first session. Follow-up was completed on 30 April 2026, the documented date of the last six-month assessment (the six-month visit of the last enrolled participant). Outcomes are reported at the scheduled visits. *Location:* Main 2.1, p. 3 (recruitment and follow-up dates)
23b	Reason trial ended	Follow-up was completed as planned: the documented closure date is 30 April 2026, the six-month assessment of the last enrolled participant (1 October 2025), with visits timed from the first treatment session. *Location:* Main 2.1, pp. 2–3
24a	Treatment delivery	Figure 1 gives delivered sessions by arm for the 44 followed for outcomes. All 44 completed three sessions. Of the seven who left, five had completed all three sessions before exclusion and two had received one session. *Location:* Main 2.4, pp. 3–4; Main 3.1, pp. 6–7
24b	Concomitant care	Both arms received the leaflet in Supplementary Methods S1 after the final session. Participants were instructed to perform it once daily, four to five days per week for four weeks, with the accompanying audiovisual material. We did not progress the dose. Both arms were telephoned to arrange appointments and to record each participant's own statement of compliance; those calls were completed for the 24 constant and 19 ascending participants in the clinical analysis. That statement was not a count of completed home sessions, and we did not analyze it as an adherence rate. We did not measure home-session completion. No interruptions or modifications of the home exercises were documented in those participants. We report NSAID use as recorded. The follow-up form asked about NSAID use, and we did not ask about splints, other analgesics, injections or additional therapies. *Location:* Main 2.4, pp. 3–4; Main 2.6, p. 5
25	Baseline characteristics	The baseline table describes the 43-person clinical population by arm and SMD. Table S1 gives the categorical Bland distribution and the comparison with age 30-65 years and Bland grades 1-3. Similarity of some variables is not used to guarantee allocation integrity. *Location:* Table 2, p. 7; Main 3.1, pp. 6–7
26	Outcomes and denominators	Figure 2 shows NPRS and BCTQ trajectories and contrasts. Table 3 reports the unadjusted pain and BCTQ contrasts; Table S4 reports their multiplicity-adjusted contrasts. Longitudinal grip outcomes have been excluded; baseline grip remains in Table 2. Table S5: pain covariance and ANCOVA sensitivity. Table S7: conservative BCTQ. Descriptive pooled results and planning scenarios are supplementary. Supplementary Tables S2 and S6 contain only NPRS/BCTQ thresholds and descriptive outcomes; no longitudinal grip results are retained. *Location:* Table 2, p. 7; Table 3, p. 8; Figure 2, p. 9; Main 3.3, p. 9

Continued on next page

Item	Reporting topic	Trial information and location (continued)
27	Adverse events	Four participants with pain and two with erythema per arm among those followed for outcomes, with overlap yielding four unique affected per arm (4/24 and 4/20). The pain was mild and the erythema was associated with the procedure; both were transient, and no treatment was given. No deaths or serious adverse events were reported among the 44 participants with available safety follow-up. Three carpal tunnel releases occurred among seven randomized participants who had each received at least one session; the orthopedics department indicated those releases early in the course (within two weeks of the participant's first treatment session, per the logbook), and they left the protocol for that reason. The three carpal tunnel releases are reported as intercurrent clinical events; causality and seriousness were not formally adjudicated, and these data neither establish nor exclude a relationship with radial pressure wave therapy. The indication was part of the institution's multidisciplinary care, outside the study protocol, and the study team did not control it. Arm, operation date and a session count for these three alone are not reported, because the arm counts and the session counts of the seven are separate totals. This report includes no safety assessment after they left the protocol.<br>*Location:* Main 3.5, p. 10
28	Ancillary analyses	Pooled trajectories and thresholds remain descriptive, future sample scenarios are in Table S3, and all additional revision analyses are explicitly exploratory. No new machine-learning model is fitted.<br>*Location:* Main 3.3, p. 9; Main 3.4, pp. 9–10
29	Interpretation	Pain is prioritized. Mean pain improved in both arms. Conclusions are inconclusive about superiority/equivalence, distinguish complete regimens from modulation alone. Pooled improvement cannot establish RPWT efficacy. Retention and tolerability provide exploratory feasibility information; no prospective success criteria were specified and feasibility was not established. Surgical indications are not assigned to a regimen because a verified reason-by-arm cross-tabulation is unavailable.<br>*Location:* Main 4, pp. 10–12; Main 5, p. 12
30	Limitations	Limitations include imprecision, post hoc endpoint-time selection, exclusion of seven participants who left the study (one constant, six ascending), baseline Bland grades above 3, unblinding, that we did not measure home-session completion. Reanalysis does not repair these limitations.<br>*Location:* Main 4, p. 12

File S2. TIDieR intervention checklist

Trial: NCT07070661. Constant versus ascending pressure–frequency RPWT regimens in carpal tunnel syndrome.

Reporting framework: Pilot exploratory report, reframed retrospectively in Round 2; randomized allocation is described in the manuscript.

Item numbering follows the source guideline; topic labels are abbreviated. Responses describe the trial and identify the relevant manuscript sections. Unavailable methodological details are stated explicitly.

Source: Hoffmann TC, Glasziou P, Boutron I, et al. Better reporting of interventions: template for intervention description and replication (TIDieR) checklist and guide. *BMJ* 2014;348:g1687. doi:10.1136/bmj.g1687. EQUATOR TIDieR checklist.

Item	Reporting topic	Trial information and location
1	Intervention name	Constant versus ascending pressure-frequency RPWT regimens, each with a common home exercise prescription.<br>*Location:* Main 2.4, pp. 3–4
2	Rationale	The comparison estimates differences between complete constant and ascending pressure/frequency schedules with an equal nominal pressure-impulse product; it does not isolate sequencing or establish acoustic energy equivalence.<br>*Location:* Main 1, p. 2; Main 2.4, pp. 3–4
3	Materials	BTL-6000 RSWT, Mexican registration 1163E2023 SSA. Supplementary Methods S1 reproduces the leaflet. Participants also received the accompanying audiovisual material.<br>*Location:* Main 2.4, pp. 3–4
4	Procedures	Fixed-point application perpendicular to the median nerve over the carpal tunnel, seated participant, supported forearm and slight wrist extension. Each session used 2500 impulses. When both wrists were affected, the more symptomatic wrist was treated and assessed (9 participants: 4 constant, 5 ascending; between-wrist pain difference 4 to 7 points). The home programme was taught for that wrist and is reproduced in Supplementary Methods S1.<br>*Location:* Main 2.2, p. 3; Main 2.4, pp. 3–4; Supplementary Methods S1
5	Provider	RPWT was delivered by a physician in the rehabilitation service who did not assess outcomes.<br>*Location:* Main 2.4, pp. 3–4
6	Mode of delivery	Individual in-person device treatment. After the final session the same leaflet was taught in person and issued with the accompanying audiovisual material. We did not individualize the programme. Both arms were telephoned to arrange appointments and to record each participant's own statement of compliance; those calls were completed for the 24 constant and 19 ascending participants in the clinical analysis. Participants were then instructed to perform the four-week programme at home.<br>*Location:* Main 2.4, pp. 3–4
7	Location	Department of Physical Medicine and Rehabilitation, Antiguo Hospital Civil de Guadalajara, Mexico; the subsequent four-week exercise programme was prescribed for completion at home.<br>*Location:* Main 2.1, pp. 2–3; Main 2.4, pp. 3–4
8	Schedule and dose	Three weekly sessions. Constant: 2500 impulses at 2 bar/10 Hz. Ascending: 800 at 1.5 bar/5 Hz, 900 at 2 bar/10 Hz, 800 at 2.5 bar/12 Hz. Nominal product 5000 bar-impulses each; theoretical emission 250.0 versus 316.7 s. Home programme: once daily on four to five days per week for four weeks. Repetitions and hold times are in Supplementary Methods S1.<br>*Location:* Main 2.4, pp. 3–4
9	Tailoring	Arm-specific RPWT settings were fixed. The home programme was the same fixed leaflet in both arms, and we did not progress it from week to week.<br>*Location:* Main 2.4, pp. 3–4
10	Modifications	No interruptions or modifications of the home programme were documented in the 24 constant and 19 ascending participants in the clinical analysis. Our leaflet is the programme in Supplementary Methods S1.<br>*Location:* Main 2.4, pp. 3–4; Main 3.1, pp. 6–7
11	Planned adherence/fidelity assessment	Session attendance was the analyzed exposure. The same calls recorded each participant's own statement of compliance for the 24 constant and 19 clinical-analysis participants. That statement was not a count of completed home sessions, and we did not analyze it as an adherence rate.<br>*Location:* Main 2.4, pp. 3–4; Main 3.1, p. 6
12	Actual adherence/fidelity	All 44 participants followed for outcomes completed three sessions. Of the seven who left, five had completed all three sessions before exclusion and two had received one session. We did not measure home-session completion.<br>*Location:* Main 3.1, p. 6; Main 4, p. 11
